# Immunological characterization and diagnostic models of RNA N6-methyladenosine regulators in Alzheimer's disease

**DOI:** 10.1038/s41598-023-41129-x

**Published:** 2023-09-04

**Authors:** Yuan Hui, Qi Ma, Xue-Rui Zhou, Huan Wang, Jian-Hua Dong, Li-Na Gao, Tian Zhang, Yan-Yi Li, Ting Gong

**Affiliations:** 1 School of Integrative Medicine, Gansu University of Traditional Chinese Medicine, Lanzhou, China; 2 Department of Encephalopathy II, Gansu Provincial Hospital of Traditional Chinese Medicine, Lanzhou, 730050 China

**Keywords:** Data mining, High-throughput screening, Machine learning

## Abstract

Alzheimer's disease (AD) is the most prevalent form of dementia, and it displays both clinical and molecular variability. RNA N6-methyladenosine (m6A) regulators are involved in a wide range of essential cellular processes. In this study, we aimed to identify molecular signatures associated with m6A in Alzheimer's disease and use those signatures to develop a predictive model. We examined the expression patterns of m6A regulators and immune features in Alzheimer’s disease using the GSE33000 dataset. We examined the immune cell infiltration and molecular groups based on m6A-related genes in 310 Alzheimer's disease samples. The WGCNA algorithm was utilized to determine differently expressed genes within each cluster. After evaluating the strengths and weaknesses of the random forest model, the support vector machine model, the generalized linear model, and eXtreme Gradient Boosting, the best machine model was selected. Methods such as nomograms, calibration curves, judgment curve analysis, and the use of independent data sets were used to verify the accuracy of the predictions made. Alzheimer's disease and non-disease Alzheimer's groups were compared to identify dysregulated m6A-related genes and activated immune responses. In Alzheimer's disease, two molecular clusters linked to m6A were identified. Immune infiltration analysis indicated substantial variation in protection between groups. Cluster 1 included processes like the Toll-like receptor signaling cascade, positive regulation of chromatin binding, and numerous malignancies; cluster 2 included processes like the cell cycle, mRNA transport, and ubiquitin-mediated proteolysis. With a lower residual and root mean square error and a larger area under the curve (AUC = 0.951), the Random forest machine model showed the greatest discriminative performance. The resulting random forest model was based on five genes, and it performed well (AUC = 0.894) on external validation datasets. Accuracy in predicting Alzheimer's disease subgroups was also shown by analyses of nomograms, calibration curves, and decision curves. In this research, we methodically outlined the tangled web of connections between m6A and AD and created a promising prediction model for gauging the correlation between m6A subtype risk and AD pathology.

## Introduction

In terms of both prevalence and impact, Alzheimer's disease (AD) stands alone as the leading cause of dementia worldwide^1^. Currently, there are an estimated 6.5 million Americans aged 65 and up who are coping with Alzheimer's disease. Unless there are significant medical advances to prevent, slow, or cure AD by 2060, this figure could reach 13.8 million^2^. As the elderly population grows, so will the fiscal and social impacts of Alzheimer's disease, according to the current paradigm of AD epidemiology^3^. As a result of AD's clinical heterogeneity and the complexity of its pathological types, no effective approach has been demonstrated to prevent the occurrence of AD, and the disease remains poorly treated^4^. More research into the causes of AD's onset and development is necessary before effective treatment options can be devised.

N6-methyladenine (m6A) modification has made great strides from prokaryotes like bacteria to eukaryotes like humans thanks to the rapid creation of specific antibodies and high-throughput sequencing^5^. It has been discovered that messenger RNAs (mRNAs), transfer RNAs (tRNAs), ribosomal RNAs (rRNAs), circular RNAs (circRNAs), microRNAs (miRNAs), and long non-coding RNAs (lncRNAs) all contain m6A modifications that serve as post-transcriptional regulatory indicators^6^. Researchers have found that m6A serves as a biomarker for control that is both dynamic and reversible, requiring both methyltransferase and demethylase to function properly. Methyltransferase-like protein 3 (METTL3), METTL14, and Wilms' tumor 1-associated protein are examples of methyltransferases, while AlkB ortholog 5 and obesity-associated protein are examples of demethylases^7–10^. The emerging field of study of m6A RNA methylation has the potential to shed new light on the mechanisms behind neural development and neurological disorders^11^. As a result, it seems fair to assume that m6A plays a significant role in AD progression. However, the processes by which m6A might be regulated in AD are currently unknown and need to be investigated further. For this reason, expanding our understanding of the molecular features of m6A regulators may shed light on the root cause of AD's pronounced variation.

## Materials and methods

### Data collection

For screening in the GEO database (GEO, https://www.ncbi.nlm.nih.gov/geo), we used the gene expression profile of human prefrontal cortex brain tissue as a criterion in order to better distinguish between healthy populations and AD patients. Two databases, GSE33000 and GSE122063, were screened using the "GEOquery" R tool. Tissue samples from the cortex of 157 healthy individuals (aged 22–106 years) and 310 individuals with Alzheimer's disease (aged 53–100 years) were chosen from the GSE33000 collection (GPL4372 platform). For validation analysis, the GSE122063 dataset (GPL16699 platform) was used, which had cortex tissues from 44 normal (ages 60–91) samples and 56 AD (ages 63–91) samples. We first selected 26 m6A RNA methylation regulators from previously published articles (Table S1). The methods used to identify m6A-related genes are consistent when applied to bulk RNA sequencing expression data. And we compared the expression profiles of 26 m6A regulators between AD and non-AD controls with an appropriate cut-off criterion: p-value < 0.05, to find differentially expressed genes associated with m6A.

### Assessing the infiltration of immune cells

It has been suggested that m6A modulators may be key variables in regulating the immune infiltration status of AD patients. We therefore evaluated the relationship between immune infiltration data and m6A gene expression profiles. To estimate the relative abundances of 22 types of immune cells in each sample using the processed gene expression data, the CIBERSORT algorithm (https://cibersort.stanford.edu/) was employed. For each sample, CIBERSORT calculates an inverse fold product p-value using Monte Carlo sampling. Immune cell fractions were only deemed reliable when the p value was less than 0.05. Each sample had a total of 22 immune cells. The correlation coefficients between the expression of m6A-related genes and the proportion of immune cells were examined to further corroborate the link between these genes and the immune cell properties associated with AD. The Spearman correlation coefficient indicated a statistically significant relationship when the p-value was less than 0.05.

### Unsupervised clustering of AD patients

We used the unsupervised clustering analysis ("ConsensusClusterPlus" R package) to divide the 310 AD samples into different groups based on the expression profiles of 26 m6A-related genes. We then used the k-means method with 1,000 iterations to reach a final classification. We decided that k = 9 was the most subtypes we could have, and we did a thorough analysis of the best number of clusters by looking at the CDF curve, the consensus matrix, and how consistent the cluster score was (> 0.9).

### Gene set variation analysis (GSVA) analysis

To better understand the variations in enriched gene sets across m6A clusters, we performed an enrichment study using the "GSVA" R package. For additional GSVA research, the "c2.cp.kegg.v7.4.symbols" and "c5.go.bp.v7.5.1.symbols" files were downloaded from the MSigDB website database. Looping through all pathways and biological processes and using the "limma" package to determine if the pathway is differentially expressed in different m6A typologies and obtain a t value. If the GSVA score |t value| was greater than 2, it was deemed to have been substantially modified.

### Weighted gene co-expression network analysis (WGCNA)

Using the "WGCNA" R package, a WGCNA analysis was carried out in order to determine which co-expression clusters existed. In order to ensure that the succeeding WGCNA analyses produce accurate and reliable findings, only the 25% of genes that ranked highest for variance were used in the analysis. An optimum soft power was used in the construction of a weighted adjacency matrix, which was then converted into a topological overlap matrix (TOM). Assuming a minimum module size of 100, we used the TOM dissimilarity measure (1-TOM) derived from the hierarchical clustering tree algorithm to generate modules. A different color was chosen at random for each section. Each module's eigengene reflected its overall pattern of gene expression. The significance of modules as an indicator of the relationship between modules and disease conditions was demonstrated by these modules. As defined by the literature, gene significance (GS) is the degree to which a gene is linked to a specific clinical trait.

### The development of a predictive model using multiple machine learning techniques

We used the "caret" R tools to create machine learning models such as the random forest model (RF), support vector machine model (SVM), generalized linear model (GLM), and eXtreme Gradient Boosting (XGB), all based on distinct m6A clusters. These four machine learning models have a good ability to screen marker genes. RF is a machine learning ensemble method that predicts categorization or regression using multiple, unrelated decision trees. The SVM method permits the creation of a hyperplane in the characteristic space that has the greatest possible separation between positive and negative examples. When evaluating the connection between normally distributed dependent features and categorical or continuous independent features, GLM, an expansion of multiple linear regression models, provides more leeway. XGB is a gradient-boosted tree ensemble that can quantitatively compare categorization errors and model complexity. In this study, we took into account the different clusters as the response variable, and cluster specific DEGs as the explanatory variables. There were 310 AD samples total, and they were split randomly between a training set (N = 217) and an additional verification set (N = 93). All of these machine learning models were run with their initial settings and evaluated using fivefold cross-validation, and their parameters were automatically tuned using a grid search by the caret package. Four machine learning models were executed, and their interpretation, residual distribution, and feature significance were visualized using the "DALEX" package. The area under the ROC curve was plotted using the "pROC" R program. Combining accuracy, precision, and recall, the best machine learning model was selected, and the top five variables were considered the most important predictive genes for Alzheimer's disease.

### Construction and validation of a nomogram model

With the help of the "rms" R package, a nomogram model was developed in order to analyze the prevalence of AD clusters. Each of these predictors has a score that corresponds to it, and the "total score" is the aggregate of all of the scores associated with the above predictors. In order to evaluate the accuracy of the nomogram model's forecasting capabilities, we made use of both the calibration curve and the DCA. Better predictive efficacy from the nomogram and a calibration curve closer to the 45° line led to a greater prediction effect. The net benefit of the model is also observed in the threshold probability interval of 0–1 to determine the predictive performance of the DCA.

### Independent validation analysis

With the help of an external brain tissue dataset, GSE122063, the ROC analyses were used to test the prediction model's ability to tell the difference between AD and non-AD controls. Again, we used gene expression profiles of human prefrontal cortex brain tissue as a standard to better distinguish between healthy populations and AD patients. Utilizing the "pROC" R software allowed for the visualization of ROC curves. In addition, we carried out the spearman correlation analysis so that we could investigate the relationships that may exist between prediction model-related genes and clinical characteristics. The cutoff for statistical significance was set at p < 0.05.

### Ethical Statement

The authors are accountable for all aspects of the work in ensuring that questions related to the accuracy or integrity of any part of the work are appropriately investigated and resolved.

## Results

### The m6A regulator landscape in Alzheimer's disease

**Figure 1:**
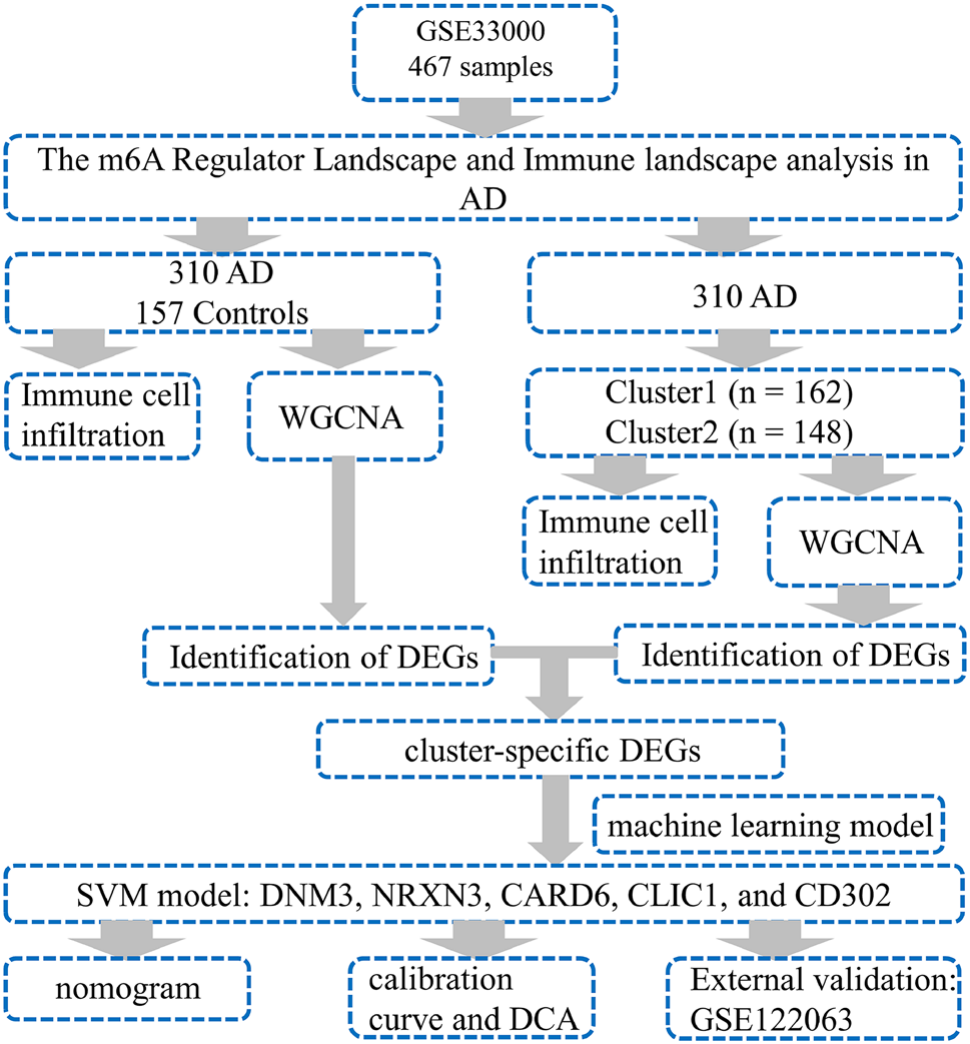
Flow chart of this study.

**Figure 2:**
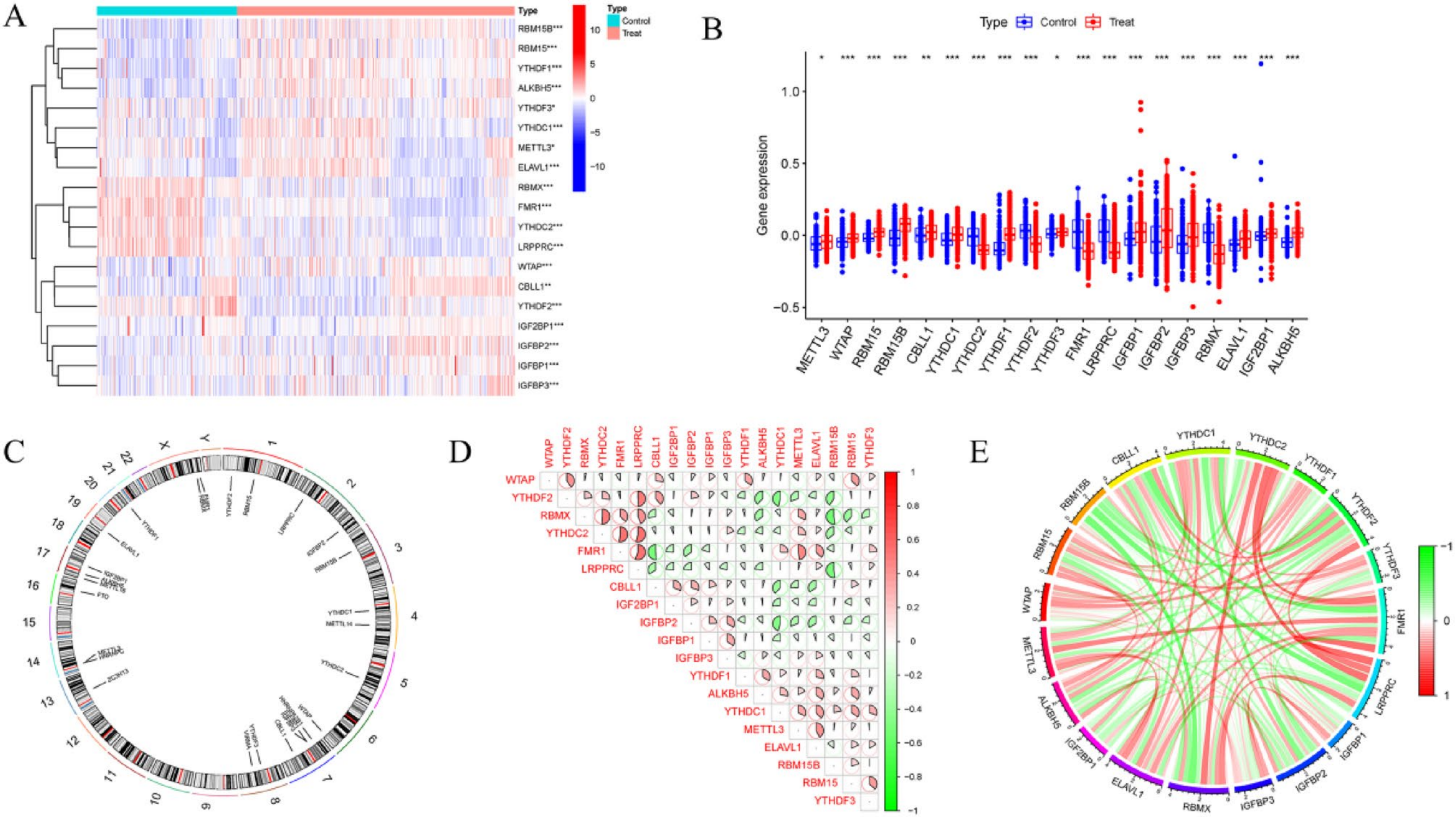
The m6A REGULATOR LANDSCAPE in Alzheimer's disease. (**A**) The heatmap displayed the expression profiles of 26 m6A regulators. (**B**) 19 m6A regulators' expression was compared between AD and non-AD samples using boxplots. *p < 0.05, **p < 0.01, ***p < 0.001. (**C**) The chromosomal localization of 26 m6A regulators. (**D**,**E**) Analysis of correlations between 19 differentially expressed m6A regulators.

The workflow of the study is outlined in Fig. 1. Using the GSE33000 dataset, we first carefully compared the expression profiles of 26 m6A regulators in AD and non-AD controls. We did this to learn more about the biological roles these regulators play in the development and progression of AD. There were a total of 19 m6A regulators that were found to be differentially expressed among the m6A transcripts. Gene expression for METTL3, WTAP, RBM15, RBM15B, CBLL1, YTHDC1, YTHDF1, YTHDF3, IGFBP1, IGFBP2, IGFBP3, ELAVL1, IGF2BP1, and ALKBH5 was higher in AD cortex samples compared to non-AD controls, while expression for YTHDC2, YTHDF2, FMR1, LRPPRC, and RBMX was generally lower (Fig. 2A,B). Using the "RCircos" program, the locations of the 26 m6A regulators on chromosomes were displayed (Fig. 2C). Subsequently, we conducted a correlation study between these m6A regulators and differential expression to inquire into whether m6A regulators played a crucial role in the development of AD. Surprisingly, some of the m6A regulators, like FMR1 and LRPPRC, as well as YTHDC2 and LRPPRC, exhibited a potent synergistic impact. Meanwhile, RBM15B and RBMX displayed behaviors that suggested they were competing with one another (Fig. 2D,E).

### Immune landscape analysis

**Figure 3:**
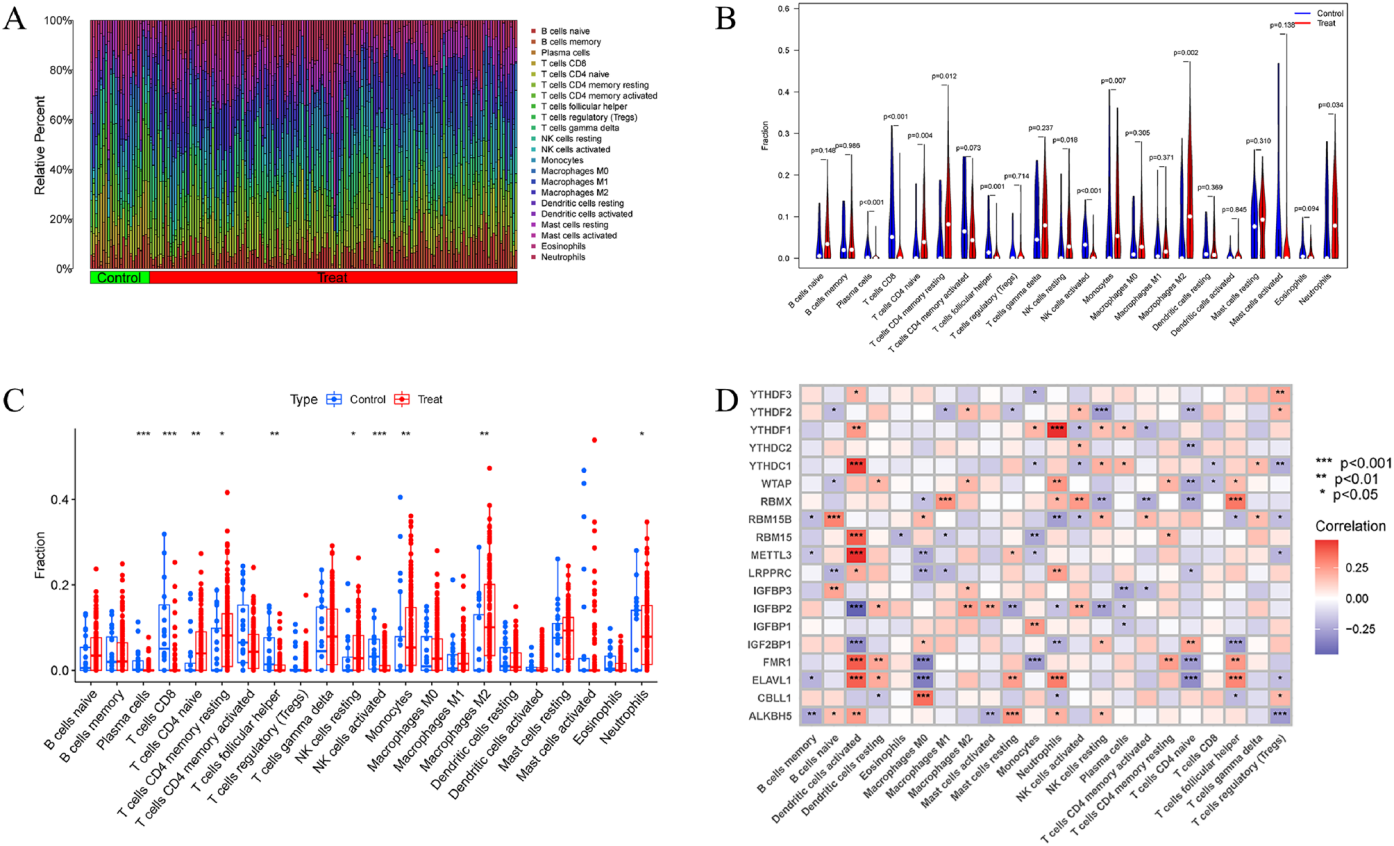
Immune landscape analysis. (**A**–**C**) Differences in immune infiltration between AD and non-AD controls. *p < 0.05, **p < 0.01, ***p < 0.001. (**D**) Analysis of the relationship between infiltrating immune cells and 19 differently expressed m6A regulators.

Using the CIBERSORT algorithm, we analyzed immune infiltration to see if there were differences in the percentages of 22 infiltrated immune cell types between the AD and non-AD groups. Results showed that AD patients had greater infiltration of T cells CD4 + naive, T cells CD4 + memory at rest, NK cells at rest, Monocytes, and Macrophages M2, indicating that immune system changes may play a significant role in the development of AD (Fig. 3A–C). In the meantime, the findings of the association analysis suggested that m6A modulators were associated with naive B cells, activated Dendritic cells, Macrophages M0 and M1, Neutrophils, and follicular helper T cells (Fig. 3D). Based on these findings, it seems likely that m6A regulators are the key variables that are responsible for regulating the molecular and immune infiltration status of AD patients.

### Identifying m6A clusters and immune infiltration similarities between m6A clusters

**Figure 4:**
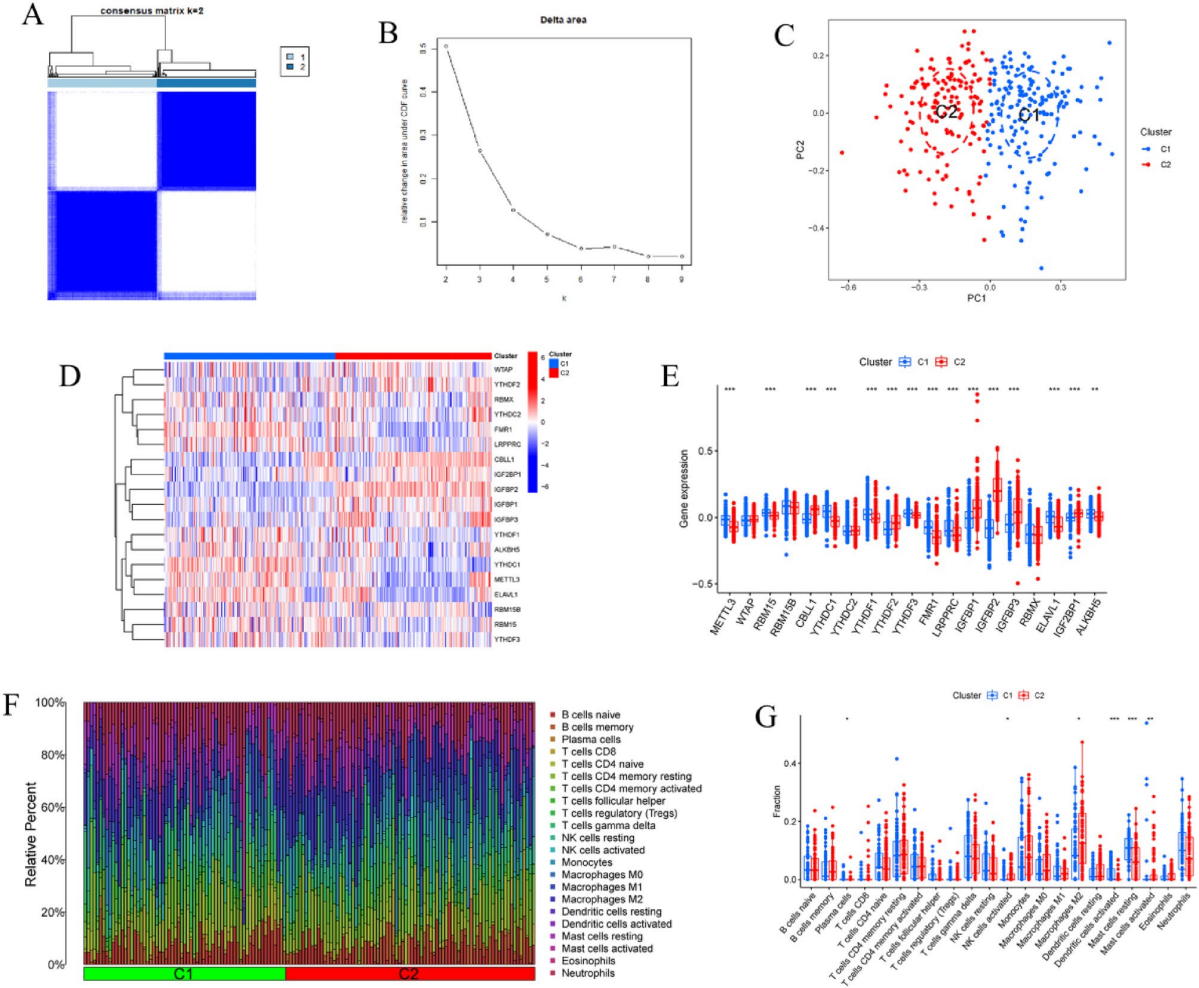
Identification of m6A-related molecular clusters in AD. (**A**) Consensus clustering matrix when k = 2. (**B**) The score of consensus clustering. (**C**) PCA analysis. (**D**) The heatmap displayed differential expression of 19 m6A regulators between the two clusters. (**E**) The expression of 19 m6A regulators was displayed in boxplots between two clusters. ***p < 0.001, **p < 0.01. (**F**) The relative proportions of 22 infiltrated immune cells between two clusters. (**G**) The disparities in immune infiltration between two clusters were depicted using boxplots. *p < 0.05, **p < 0.01 ***p < 0.001.

We used a method called consensus clustering to put the 310 AD samples into groups based on the expression profiles of 26 m6A regulators. This helped us learn more about how m6A affects expression in AD. When k was set to 2, the cluster values were the most consistent (Fig. 4A,B). Principal component analysis revealed that the two m6A groups had different rates of transcription (Fig. 4C). Using the agreement matrix heatmap in conjunction with the data, we were able to divide the 310 AD patients into two distinct groups: Cluster 1 (n = 162) and Cluster 2 (n = 148) (Fig. 4D). The genes METTL3, RBM15, YTHDC1, YTHDF1, YTHDF3, FMR1, LRPPRC, ELAVL1, and ALKBH5 were overexpressed in m6A Cluster 1, while CBLL1, YTHDF2, IGFBP1, IGFBP2, IGFBP3, and IGF2BP1 were highly expressed in m6A Cluster 2 (Fig. 4E). Analysis of immune infiltration also revealed differences in the immunological microenvironment between m6A Clusters 1 and 2 (Fig. 4F). Dendritic cells and mast cells were more prevalent in Cluster 1, while NK cells, M2 macrophages, and activated mast cells were more common in Cluster 2 (Fig. 4G).

### GSVA functional analysis

**Figure 5:**
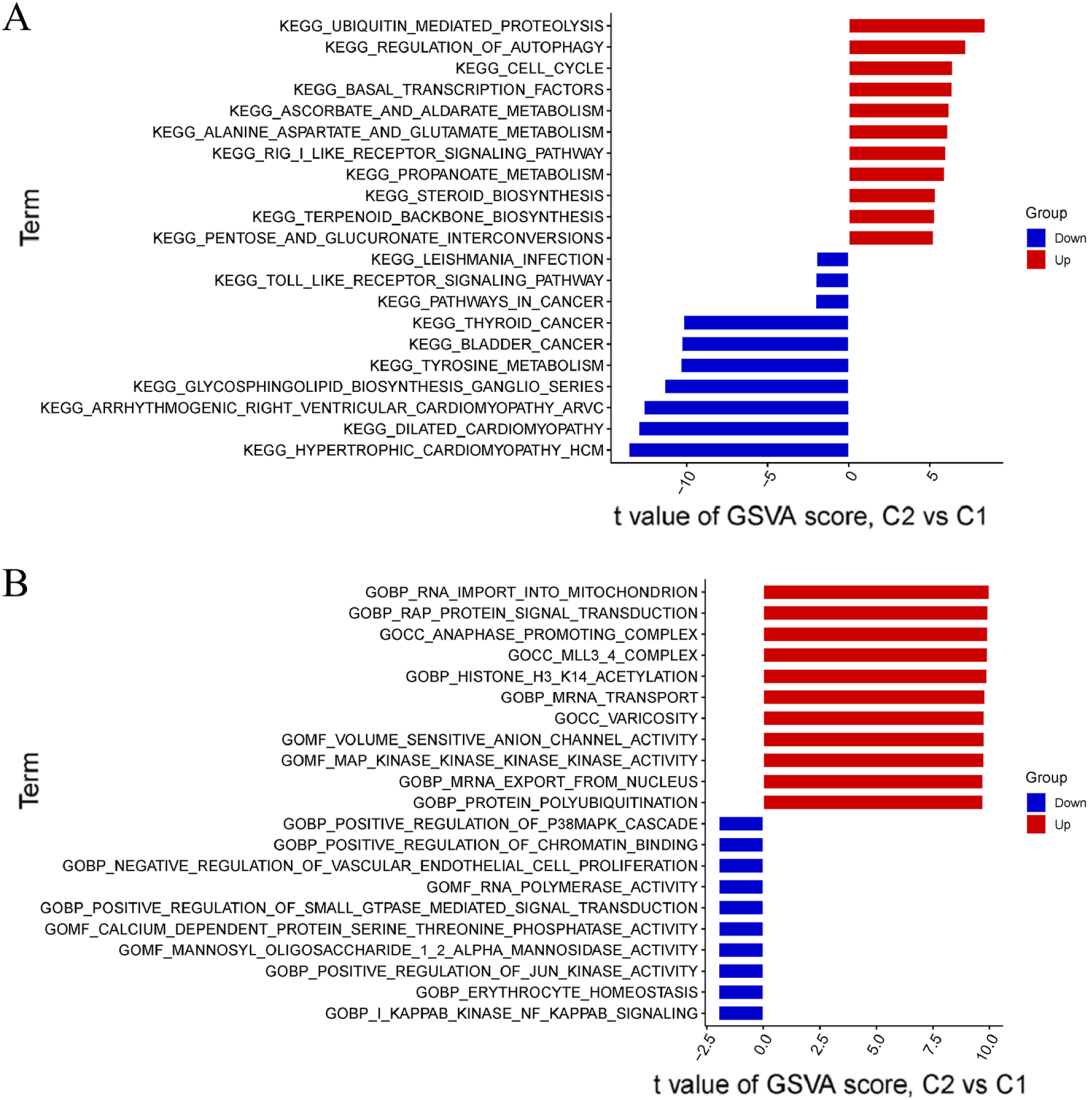
GSVA functional analysis. (**A**) Variations in the levels of activity found within hallmark pathways between Cluster 1 and Cluster 2. (**B**) Differences in the biological functions exhibited by samples from Cluster 1 and Cluster 2 respectively.

To learn more about the functional distinctions between the two groups of m6A regulators, the GSVA analysis was employed. Cluster 1 showed upregulation of genes involved in the Toll-like receptor signaling pathway, leishmania infection, and many cancers; Cluster 2 showed upregulation of genes involved in ubiquitin-mediated proteolysis, cell cycle control, and autophagy (Fig. 5A). Cluster 2 upregulated protein polyubiquitination, mRNA export from the nucleus, and mRNA transport, while Cluster 1 was significantly associated with the regulation of the positive regulation P38MAPK cascade, Positive regulation of chromatin binding, and RNA polymerase activity (Fig. 5B).

### The identification of gene modules and the building of co-expression networks

**Figure 6:**
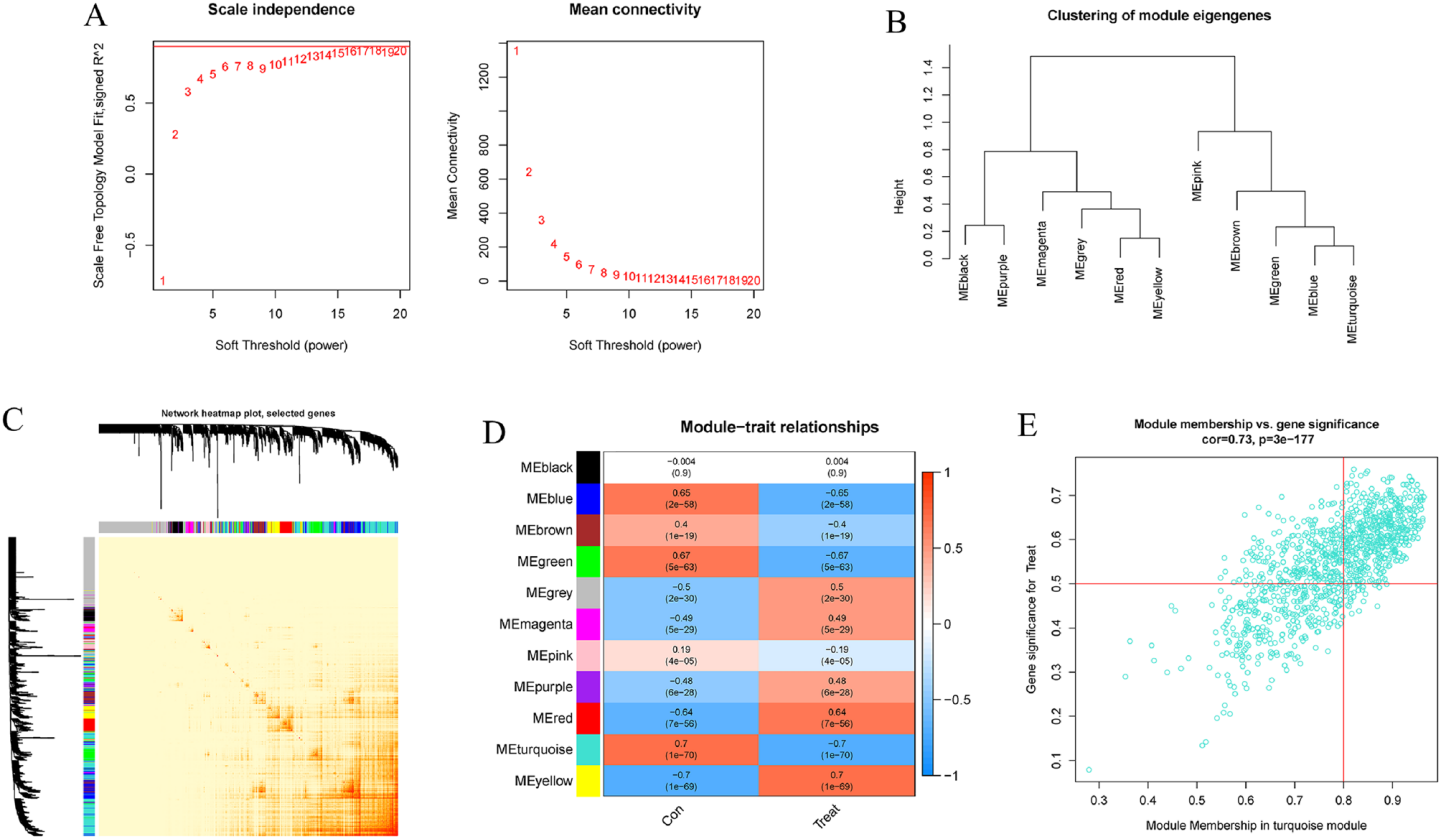
Co-expression network in AD of genes with differential expression. (**A**) The selection of power with a flexible threshold. (**B**) Module genes clustering representation. (**C**) A heatmap depicting the correlations between 11 modules. (**D**) Analysis of the correlation between module genes and clinical status. (**E**) Scatter diagram depicting the relationship between module membership in the turquoise module and the genetic significance of AD.

We used the WGCNA method to make a co-expression network and modules for the normal and AD participants in order to find the important gene modules linked to AD. We determined the variance in expression for each gene in GSE33000 and then focused on the top 25% of genes by variance. When the soft power was 15, the scale-free R^2^ was 0.90, and co-expressed gene modules were found (Fig. 6A). The dynamic cutting approach was used to acquire 10 colored co-expression modules, and a heatmap of the topological overlap matrix (TOM) was also shown (Fig. 6B,C). Following this, the co-expression of these genes across the 10 color modules and their associated clinical features (Control and Treat) were continually analyzed. Last but not least, 759 genes in the turquoise module showed the highest association with AD (Fig. 6D). Additionally, we found that the turquoise module was positively correlated with genes involved in other modules (Fig. 6E).

**Figure 7:**
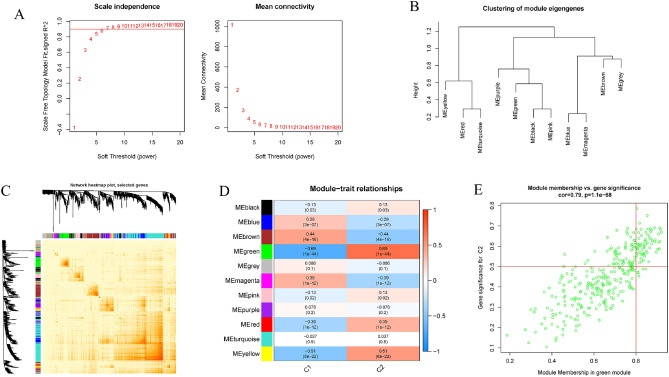
Co-expression network of genes that differ in expression between the two clusters. (**A**) The selection of power with a flexible threshold. (**B**) Module genes clustering representation. (**C**) A heatmap depicting the correlations between 11 modules. (**D**) Analysis of the correlation between module genes and clinical status. (**E**) Scatter diagram depicting the relationship between module membership in the green module and gene significance for cluster2.

We also used the WGCNA approach to examine the most important gene modules that are in close proximity to m6A clusters. The best soft threshold values for building a scale-free network were β = 6 and R^2^ = 0.9, according to our screening. (Fig. 7A). Ten color-coded co-expression modules were extracted using the dynamic cutting technique and the topological overlap matrix heatmap (Fig. 7B,C). The substantial association between the green module (424 genes) and AD clusters was revealed by analyzing the relationships between modules and clinical characteristics (Cluster1 and Cluster2) (Fig. 7D). A correlation study indicated a strong association between green module genes and the target module (Fig. 7E).

### Building models using machine learning and evaluating their performance

**Figure 8:**
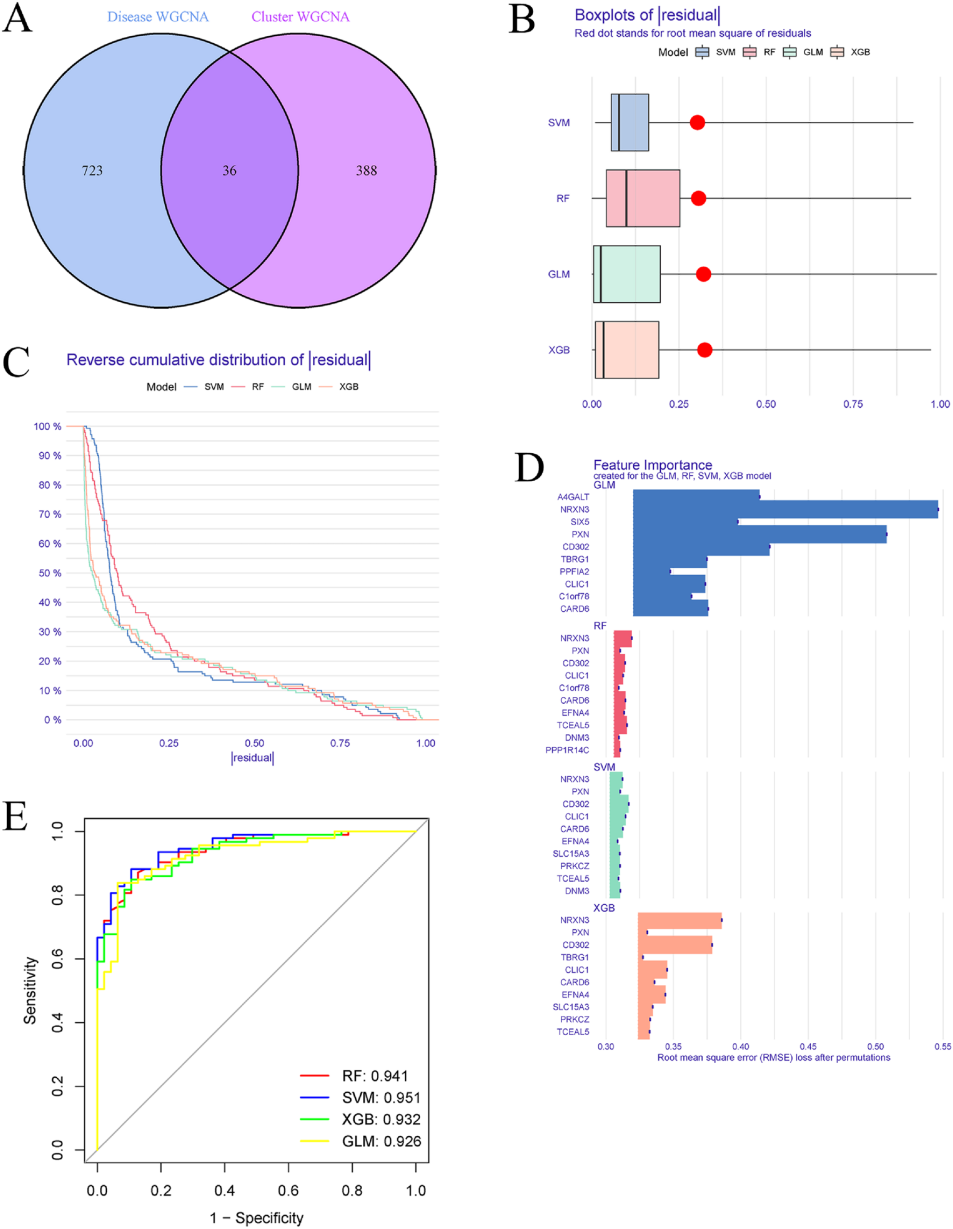
Machine learning models using RF, SVM, GLM, and XGB are built and evaluated. (**A**) The intersections of module-related genes from m6A clusters and module-related genes from the GSE33000 dataset. (**B**) Boxplots illustrated each machine learning model's residuals. The red dot signified the root mean square of residuals (RMSE). (**C**) Distribution of cumulative residuals for each machine learning model. (**D**) The salient characteristics of the RF, SVM, GLM, and XGB models of machine learning. (**E**) Four machine learning models were tested using a fivefold cross-validation procedure, and the results were analyzed using the ROC curve.

By comparing the genes related to the AD and non-AD modules to the genes related to the m6A cluster modules, 36 cluster-specific DEGs were found (Fig. 8A). Based on the expression profiles of the 36 cluster-specific DEGs in the AD training cohort, we set up four proven machine learning models [random forest model (RF), support vector machine model (SVM), general linear model (GLM), and eXtreme Gradient Boosting (XGB)] to find more subtype-specific genes with high diagnostic value. To explain the four models and visualize the residual distribution for each model in the test set, the "DALEX" package was used. The residual variance in SVM and RF machine learning models was relatively low (Fig. 8B,C). After that, the root mean square error (RMSE) was used to determine the order of importance of each model's 15 most salient feature variables (Fig. 8D). In addition, we calculated receiver operating characteristic (ROC) curves based on fivefold cross-validation to assess the discriminative performance of the four machine learning algorithms in the testing set. The ROC area under the curve (AUC) was highest for the RF machine learning model (SVM, AUC = 0.951; RF, AUC = 0.941; XGB, AUC = 0.932; GLM, AUC = 0.926, Fig. 8E). Taken together, these findings show that the SVM model is superior for distinguishing between patient groupings. After running the SVM model, the top five variables (DNM3, NRXN3, CARD6, CLIC1, and CD302) were chosen as predictor genes.

**Figure 9:**
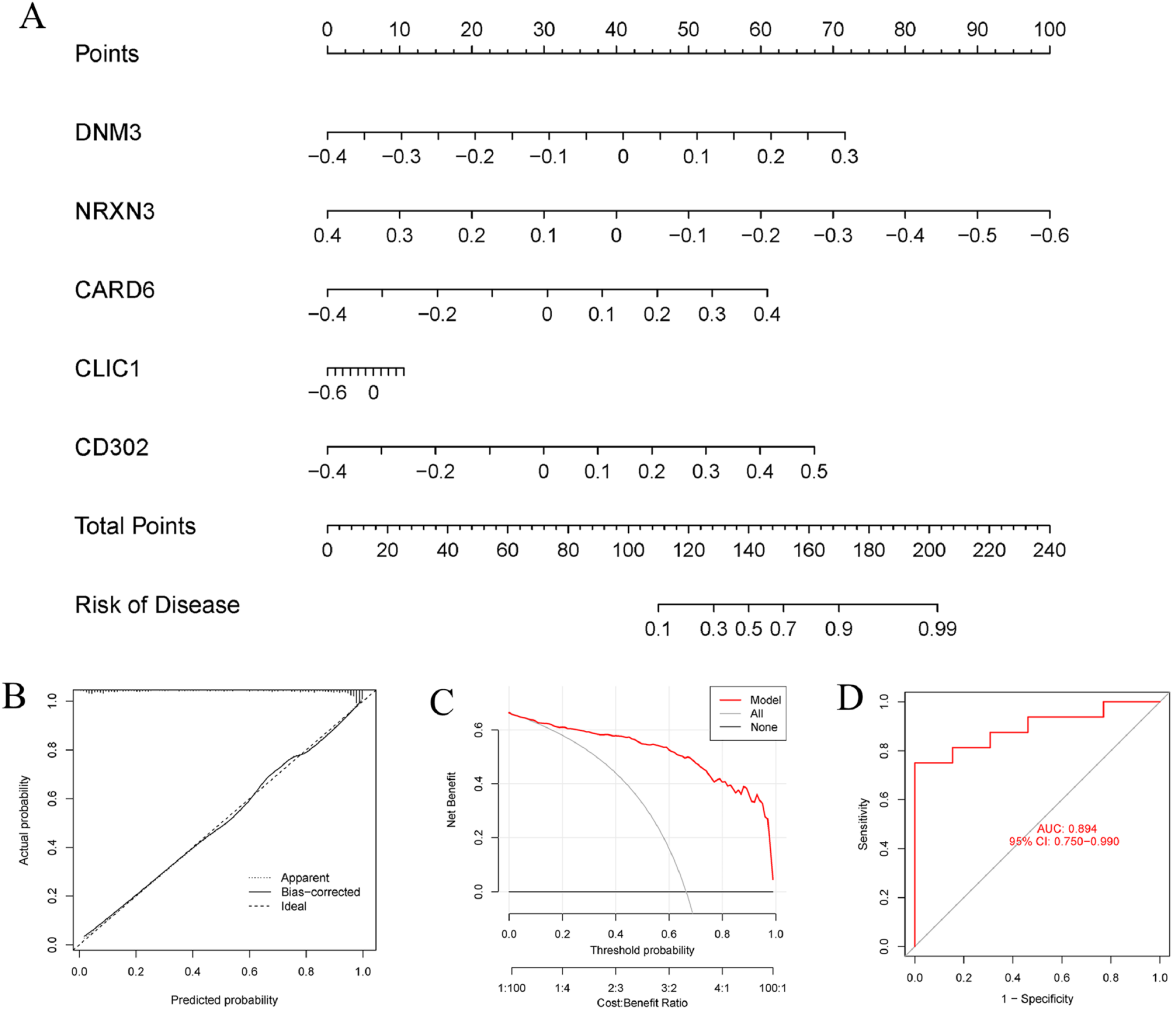
The validation of the five-gene SVM model. (**A**) Using the 5-gene SVM model, a nomogram was constructed to predict the risk of AD clusters. (**B**) Nomogram model predictive efficacy evaluation by calibration curve. (**C**) A discriminant analysis was used to evaluate the nomogram's sensitivity to change. (**D**) ROC analysis of the 5-gene-based SVM model in GSE122063 datasets.

To see how well the SVM model could predict, we first made a nomogram (Fig. 9A) to predict the likelihood of m6A clusters in 310 AD patients. The nomogram model's predictive efficacy was calculated using the calibration curve and decision curve analysis (DCA). The calibration curve and discriminant analysis (DCA) both show that our nomogram is very accurate and may serve as a foundation for clinical decision-making regarding AD clusters (Fig. 9B,C). Our 5 gene prediction algorithm was then tested on a single external brain tissue dataset consisting of both healthy individuals and AD patients to ensure its accuracy. The ROC curves for the GSE122063 dataset showed that the 5-gene prediction model performed satisfactorily, with an AUC value of 0.894, indicating that our diagnosis model is similarly successful in identifying AD in normal individuals (Fig. 9D).

**Figure 10:**
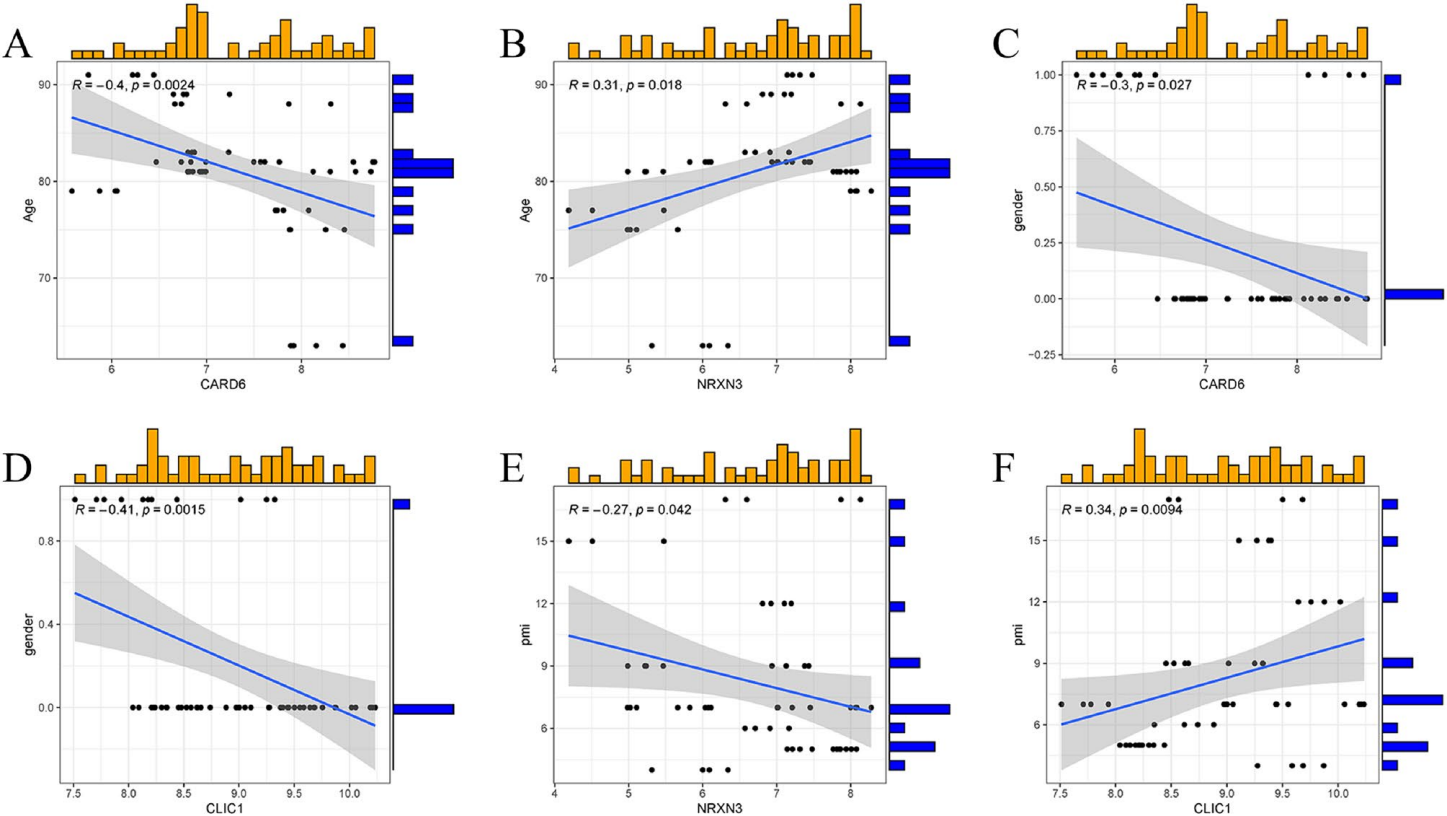
Use of the GSE122063 dataset to verify the accuracy of correlation analysis. (**A**,**B**) The association between CARD6, NRXN3, and age. (**C**,**D**) The association between CARD6, CLIC1 and gender. (**E**,**F**) The association between NRXN3, CLIC1 and pmi.

Additionally, we used a third-party dataset (GSE122063) to verify our findings about the predictor genes' association with clinical variables. (Fig. 10A–F) While NRXN3 was positively connected with age (R = 0.31), we discovered that CARD6 was inversely correlated with age (R = − 0.4). There was an inverse relationship between gender and CARD6 and CLIC1 (R = − 0.3 for CARD6 and R = − 0.41 for CLIC1). R = − 0.27 indicated a negative relationship between NRXN3 and PMI. This finding demonstrates the superior diagnostic usefulness of the 5-gene prediction model in pathology.

## Discussion

The current treatment for AD is insufficiently effective because of the variability of AD pathophysiology^12^. The quick examination of millions of polymorphisms in thousands of participants made possible by recent breakthroughs in high-throughput genome technology has greatly improved our understanding of the genetic basis of AD susceptibility^13^. Among the many different modifications to mRNA, m6A is very important for controlling the mRNA's fate^14,15^. The possible involvement of m6A in AD is still unclear, despite previous investigations suggesting it may play a vital role in neurodegenerative diseases^16–18^. As a result, we sought to better understand how the m6A-related genes play a part in the AD phenotype and the immunological microenvironment. m6A-related gene profiles were also used for subtype prediction in AD.

For the first time, we compared normal participants and AD patients in terms of the expression profiles of m6A regulators in brain tissues. The fact that Alzheimer's disease (AD) patients are more likely than controls to have m6A regulators that are not working properly suggests that m6A regulators play a key role in the development of AD. As evidenced by the occurrence of interactions between m6A regulators in AD patients, correlation analysis revealed that several m6A modulators demonstrated strong synergistic or antagonistic effects. Consistent with these findings, a previous investigation indicated that immune cell infiltration in AD patients' blood or brain tissue was significantly higher. Infiltration of CD4(+) naive T cells, CD4(+) memory type T cells, NK quiescent cells, M219-21 monocytes, and M221 macrophages was increased in AD patients^19–21^. To further show the varied m6A regulation patterns in AD patients, we used unsupervised cluster analysis to categorize the expression landscapes of m6A regulators and found two separate m6A-related clusters. Cluster 2 has dramatically increased the expression of genes involved in ubiquitin-mediated proteolysis and autophagy control, according to GSVA. It has been demonstrated through research that impaired neuronal autophagy is a major contributor to the onset and progression of neurodegenerative illnesses like Alzheimer's disease. It is possible that autophagy's effects on AD22–27 are mediated by the fact that it plays a crucial role in the metabolism of A and tau proteins, the mTOR pathway, neuroinflammation, and the endocrine system^22–27^.

Multifactorial analyses have taken relationships between variables into account, having a lower error rate and more reliable results compared to univariate analyses, and machine learning models based on demographic and imaging metrics have been increasingly applied for the prediction of AD prevalence in recent years. Here, we established an SVM-based prediction model, that demonstrated the highest predictive efficacy in the testing cohort (AUC = 0.951), suggesting that SVM-based machine learning has satisfactory performance in predicting the subtypes of AD, based on the expression profiles of cluster-specific DEGs. Then, we built a 5-gene SVM model by selecting five key variables (DNM3, NRXN3, CARD6, CLIC1, and CD302). DNM3 is a microtubule-associated protein that functions in the formation of microtubule bundles by binding and hydrolyzing guanosine triphosphate^28^. DNM3 may be a useful target for the therapy of age-related neurodegenerative diseases, as studies have revealed that genetic variability in DNM3 alters the age of onset for LRRK2 Gly2019Ser Parkinsonism and informs disease-relevant translational neuroscience^29^. NRXN3 is a member of the neurexin (NRXN) family of proteins that plays a role in synaptogenesis and intercellular signaling in the nervous systems of vertebrates^30^. According to one study, NRXN3 downregulation is the most significant risk factor for Alzheimer's disease and ageing^31^. CARD is a homotypic protein–protein interaction module that links components of signal transduction pathways involved in the modulation of apoptosis or innate immunity^32^. CARD (CARDia-associated receptor decoy) is a homotypic protein–protein interaction module that connects parts of signal transduction pathways that regulate apoptosis and innate immunity. As a CARD family member, CARD6's initial role is hypothesised to be to activate NF-κB signaling by multiple distinct pathways^33^. RIP2, another CARD-containing protein kinase that causes NF-κB activation, interacts with CARD6^34^. Given CARD6's central function in controlling inflammation and apoptosis, we hypothesized that a shift in CARD6 expression would be linked to the onset of AD. Neurotoxicity caused by amyloid beta induced microglia has been demonstrated to be mitigated by inhibiting CLIC1. Initiating and boosting microglia ROS production, CLIC1 has a unique role in the fight against neurodegeneration in AD, making it an excellent and new therapeutic target^35^. Neurotoxicity caused by amyloid beta induced microglia has been demonstrated to be mitigated by inhibiting CLIC1. Initiating and boosting microglia ROS production, CLIC1 has a unique role in the fight against neurodegeneration in AD, making it an excellent and new therapeutic target^36^.

The 5-gene-based SVM model, which has an AUC of 0.894 in external validation datasets, provides new insights into the diagnosis of AD. Importantly, we used the DNM3, NRXN3, CARD6, CLIC1, and CD302 to create a nomogram model for the differential identification of AD subgroups. Our results showed that this model had outstanding predictive efficacy, suggesting it could be useful in clinical settings. The present study does have a few caveats, though. First, the expression levels of m6A regulators were validated in our current work based on extensive bioinformatics analysis; however, additional clinical or experimental tests are necessary to draw firm conclusions. In addition, the efficacy of the prediction model needs to be verified through more in-depth clinical characterization.

## Conclusion

When we looked at our research as a whole, we found a link between m6A regulators and infiltrated immune cells. We also learned that the immune systems of Alzheimer's disease patients with different m6A clusters are very different. A 5-gene-based SVM model was found to be the best model for machine learning because it can reliably measure AD subtypes and the pathological outcome of AD patients. Our research further elucidates the underlying molecular pathways that contribute to AD heterogeneity and identifies for the first time the function that m6A plays in the disease of Alzheimer's. However, current research is still hampered by significant limitations. To begin, there is a paucity of clinical data and experimental studies to further verify the conclusions, and all inferences are made based on the processing and analysis of data available from public databases. Furthermore, when comparing control and AD samples, m6A profiles differed significantly in different brain regions. The model we employed successfully captured the changes observed in the frontal cortex, however, this model may not provide a comprehensive representation. To further validate the model's efficacy in clinical settings, it will be necessary to gather additional AD cases and conduct a large number of prospective clinical assessments in the future.

### Supplementary Information

Supplementary Table 1.

## Data Availability

The raw data of this study is derived from the GEO (https://www.ncbi.nlm.nih.gov/geo/) which is publicly available databases.
