# Supplementary material for: Immunological characterization and diagnostic models of RNA N6-methyladenosine regulators in Alzheimer's disease
Source: Sci Rep. 2023 Sep 4;13:14588. doi: 10.1038/s41598-023-41129-x (PMC10477294; doi:10.1038/s41598-023-41129-x)
Supplement: Supplementary file 1 — Supplementary Table 1. [file 41598_2023_41129_MOESM1_ESM.docx]

Table S1 Genes associated with RNA N6-methyladenosine

| m6A regulators |
| --- |
| METTL3 |
| METTL14 |
| METTL16 |
| WTAP |
| VIRMA |
| ZC3H13 |
| RBM15 |
| RBM15B |
| CBLL1 |
| YTHDC1 |
| YTHDC2 |
| YTHDF1 |
| YTHDF2 |
| YTHDF3 |
| HNRNPC |
| FMR1 |
| LRPPRC |
| HNRNPA2B1 |
| IGFBP1 |
| IGFBP2 |
| IGFBP3 |
| RBMX |
| ELAVL1 |
| IGF2BP1 |
| FTO |
| ALKBH5 |
